# Effects of acute and chronic administration of methylprednisolone on oxidative stress in rat lungs* **

**DOI:** 10.1590/S1806-37132014000300006

**Published:** 2014

**Authors:** Ronaldo Lopes Torres, Iraci Lucena da Silva Torres, Gabriela Laste, Maria Beatriz Cardoso Ferreira, Paulo Francisco Guerreiro Cardoso, Adriane Belló-Klein

**Affiliations:** Hospital Divina Providência, Porto Alegre, Brazil; Department of Pharmacology, Institute of Basic Health Sciences, Federal University of Rio Grande do Sul, Porto Alegre, Brazil; Institute of Basic Health Sciences, Federal University of Rio Grande do Sul, Porto Alegre, Brazil; Department of Pharmacology, Institute of Basic Health Sciences, Federal University of Rio Grande do Sul, Porto Alegre, Brazil; Department of Cardiorespiratory Diseases, Thoracic Surgery Section, Heart Institute, University of São Paulo School of Medicine Hospital das Clínicas, São Paulo, Brazil; Department of Physiology. Institute of Basic Health Sciences, Federal University of Rio Grande do Sul, Porto Alegre, Brazil

**Keywords:** Lung, Methylprednisolone, Glucocorticoids, Lipid peroxidation, Antioxidant response elements

## Abstract

**Objective:**

To determine the effects of acute and chronic administration of methylprednisolone on oxidative stress, as quantified by measuring lipid peroxidation (LPO) and total reactive antioxidant potential (TRAP), in rat lungs.

**Methods:**

Forty Wistar rats were divided into four groups: acute treatment, comprising rats receiving a single injection of methylprednisolone (50 mg/kg i.p.); acute control, comprising rats i.p. injected with saline; chronic treatment, comprising rats receiving methylprednisolone in drinking water (6 mg/kg per day for 30 days); and chronic control, comprising rats receiving normal drinking water.

**Results:**

The levels of TRAP were significantly higher in the acute treatment group rats than in the acute control rats, suggesting an improvement in the pulmonary defenses of the former. The levels of lung LPO were significantly higher in the chronic treatment group rats than in the chronic control rats, indicating oxidative damage in the lung tissue of the former.

**Conclusions:**

Our results suggest that the acute use of corticosteroids is beneficial to lung tissue, whereas their chronic use is not. The chronic use of methylprednisolone appears to increase lung LPO levels.

## Introduction

Corticosteroids are extensively used in a wide range of respiratory tract disorders, such as asthma, allergic rhinitis, and COPD.^(^ ^1^ ^)^ It has been observed that acute treatment with corticosteroids can suppress inflammatory processes and reactive oxygen species (ROS) production. ^(^ ^2^ ^)^ In a recent study,^(^ ^3^ ^)^ it was shown that the administration of dexamethasone decreases lung tissue malondialdehyde production after ischemia/reperfusion injury and protects cellular levels of antioxidant enzymes. In addition, short-term administration of prednisolone or dexamethasone has been shown to inhibit ROS generation in platelets, and there is evidence that steroids inhibit oxidative phosphorylation.^(^ ^4^ ^)^ It has been suggested that the long-term use of corticosteroids at low doses (1-2 mg/kg per day) can benefit the lungs and reduce the risk of systemic side effects in patients with acute respiratory distress syndrome,^(^ ^5^ ^)^ whereas acute administration of high doses of corticosteroids has been found to produce no benefits in such patients.^(^ ^6^ ^)^

Chronic treatment with corticosteroids can induce a variety of symptoms and signs (side effects), including truncal obesity, facial swelling ("moon face"), cutaneous striae, hirsutism, cataract, osteoporosis, myopathy, diabetes mellitus, immunosuppression, and cardiovascular disorders.^(^ ^7^ ^)^ Excess corticosteroid use can also induce overproduction of ROS by endothelial cells.^(^ ^8^ ^)^

It is well known that corticosteroids have anti-inflammatory effects, some of which can be mediated by ROS, which are products of normal metabolic processes in cells. The major sources of ROS are leakages from the electron transport chain in mitochondria and endoplasmic reticulum. Another important source of ROS is a membrane-associated NADH/NADPH oxidase. At low concentrations, ROS act as physiological mediators of cellular responses and regulators of gene expression.^(^ ^4^ ^)^ The imbalance between the production of ROS and antioxidant defenses leads to oxidative stress.^(^ ^9^ ^)^ Oxidative stress has been implicated as an important pathologic factor in pulmonary, neurodegenerative, and autoimmune diseases, as well as in metabolic disorders, cancer, and aging.^(^ ^10^ ^-^ ^12^ ^)^ It is well known that ROS generate a biochemical cascade, producing lipid peroxidation (LPO), protein oxidation, DNA damage, and cell death, all of which can contribute to the occurrence of pathological conditions associated with a marked increase in ROS and other free radicals,^(^ ^13^ ^)^ such as ischemia/reperfusion-induced lung injury.^(^ ^14^ ^)^ Therefore, ROS play a crucial role in the cascade of events that lead to lung failure. 

Taking all of the above into account, we conducted the present study with the objective of determining the effect of acute and chronic administration of methylprednisolone on oxidative stress. To that end, we quantified LPO and total reactive antioxidant potential (TRAP) in rat lungs. 

## Methods

Forty experimentally naive adult (60-day-old) male Wistar rats (200-250 g) were randomized by weight and housed in groups of five in polypropylene home cages (49 × 34 ×16 cm). All animals were maintained on a standard 12/12-h light/dark cycle (lights on at 7:00 a.m. and off at 7:00 p.m.) in a temperature-controlled environment (22 ± 2°C) and were given *ad libitum* access to water and chow. All experiments and procedures were approved by the institutional animal care and use committee and were in compliance with the Brazilian guidelines involving the use of animals in research (Law no. 11,794) and with international guidelines. Vigorous attempts were made to minimize animal suffering and to decrease external sources of pain and discomfort, as well as to use only the number of animals required in order to produce reliable scientific data.

We used methylprednisolone sodium succinate (Solu-Medrol^(r)^, Pharmacia, New York, NY, USA). The lyophilized powder (500 mg) was dissolved in 8 mL of 0.9% saline solution. The drug solution was prepared immediately prior to its administration. 

In the acute treatment experiment, the animals were divided into two groups (n = 10 each). The rats in one group (the acute treatment group) received a single injection of methylprednisolone (50 mg/kg, i.p.) in a volume of 1 mL/kg of the solution, whereas those in the other group (the acute control group) were injected with an equal volume of saline (i.p.). 

In the chronic treatment experiment, the animals were divided into two groups (n = 10 each). The rats in one group (the chronic treatment group) received methylprednisolone (6 mg/kg per day, p.o.) in drinking water for 30 days, whereas those in the other group (the chronic control group) received drinking water only. Each 500 mL of the drinking water contained 31 mg of methylprednisolone sodium succinate (0.0625 mg/mL). Considering a mean consumption of 25 mL/day per rat, each chronic treatment group rat consumed 1.56 mg of methylprednisolone per day. 

At 24 h after acute administration or at the end of the chronic treatment period, the animals were killed by decapitation. The lungs were extracted and frozen by immersion in liquid nitrogen. Samples were stored at −80°C until analysis. The lungs were weighed and homogenized at 1:5 w/v in ice-cold (1.15% KCl and 20 mmol/L phenylmethylsulfonyl fluoride) fluid using an Ultra-Turrax homogenizer (IKA, Toronto, Ontario, Canada). To remove the particulate fraction, the homogenates were centrifuged at 1,000 g for 20 min at 0-4°C, and the supernatant was used for LPO, TRAP, and protein content assays.^(^ ^15^ ^)^

The level of TRAP was determined by measuring luminol chemiluminescence intensity induced by the thermolysis of 2,2'-azobis(2-amidinopropane) dihydrochloride.^(^ ^16^ ^)^ The results are expressed as µM of 6-hydroxy-2,5,7,8-tetramethylchroman-2-carboxylic acid per mg of protein. We quantified LPO using chemiluminescence. The method is highly sensitive and capable of detecting small amounts of peroxidation products. Chemiluminescence was measured in a liquid scintillation counter using the out-of-coincidence mode (LKB Rack Beta Liquid Scintillation Spectrometer 1215; LKB Produkter AB, Bromma, Sweden). The reactions were started by the addition of 3 mmol/L tert-butyl hydroperoxide, and the data are expressed as counts per second (cps) per mg of protein in the homogenate.^(^ ^17^ ^)^ Protein levels were measured with the method devised by Lowry et al.,^(^ ^18^ ^)^ and bovine serum albumin was used as the standard.

The data are expressed as mean ± SE and statistically evaluated using the Student's t-test. Values of p < 0.05 were considered significant.

## Results

**Figure 1:**
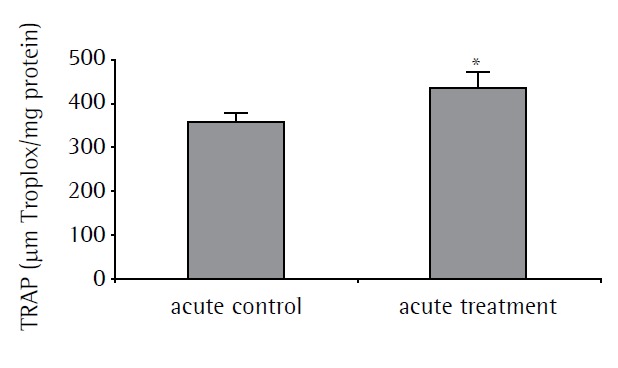
Mean levels of total reactive antioxidant potential (TRAP) in the lungs of rats subjected to acute administration of methylprednisolone (acute treatment group) or injected with an equal volume of saline (acute control group). Trolox: 6-hydroxy-2,5,7,8-tetramethylchroman-2-carboxylic acid. *p < 0.05, Student's t-test.

**Figure 2:**
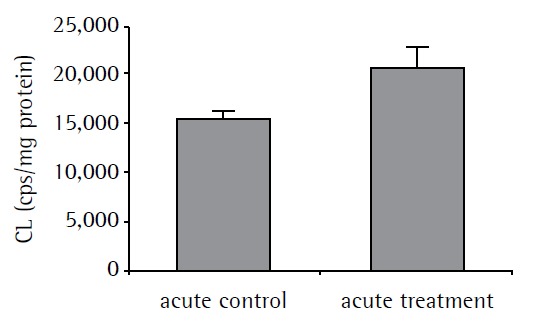
Mean levels of lipid peroxidation in the lungs of rats subjected to acute administration of methylprednisolone (acute treatment group) or injected with an equal volume of saline (acute control group). CL: chemiluminescence; and cps: counts per second.

We first evaluated the effect of acute treatment with methylprednisolone on the levels of TRAP and LPO in rat lungs. A significant (20%) increase was observed in total TRAP levels in the treated group (p < 0.05; Figure 1). No significant difference was found between the groups regarding LPO levels (p > 0.05; Figure 2).

**Figure 3:**
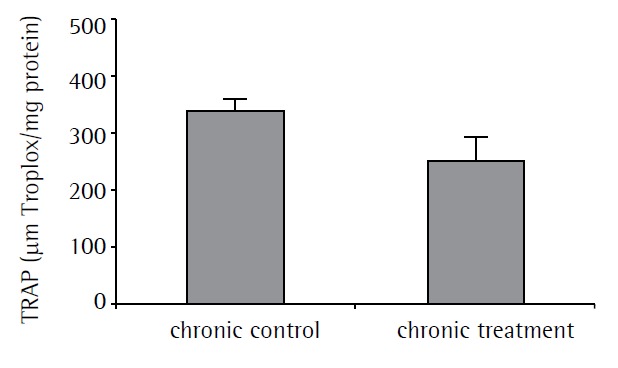
Mean levels of total reactive antioxidant potential (TRAP) in the lungs of rats subjected to chronic administration of oral methylprednisolone (chronic treatment group) or not (chronic control group). Trolox: 6-hydroxy-2,5,7,8-tetramethylchroman-2-carboxylic acid.

**Figure 4:**
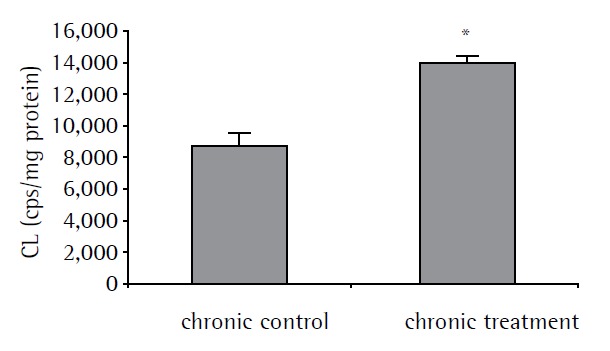
Mean levels of lipid peroxidation in the lungs of rats subjected to chronic administration of oral methylprednisolone (chronic treatment group) or not (chronic control group). CL: chemiluminescence; and cps: counts per second. *p < 0.05, Student's t-test.

We found no difference between the chronic treatment group and the chronic control group in terms of the total TRAP levels (p > 0.05; Figure 3). The degree of pulmonary oxidative damage, as assessed by chemiluminescence, was significantly (38%) greater in the chronic treatment group than in the chronic control group (p < 0.05; Figure 4).

## Discussion

Antioxidant concentrations in the lungs can be quantified by determining the level of TRAP. ^(^ ^10^ ^,^ ^14^ ^)^ The relative concentration of antioxidants determines the total tissue antioxidant capacity. The TRAP level primarily represents non-enzymatic water-soluble antioxidants in the tissue. In addition, the level of LPO, which plays an important role in the induction of free radical formation and apoptosis,^(^ ^19^ ^)^ is widely used as a marker of oxidative stress.

The results of the present study show that the duration of corticosteroid treatment alters the oxidative system responses in the lungs of rats. Acute treatment with methylprednisolone induced a significant increase in TRAP levels in rat lungs without any changes in LPO levels. However, when the treatment was maintained for 30 days, we observed an increase in LPO levels without any changes in TRAP levels, which increases the risk of oxidative lung injury. Nevertheless, when animals were submitted to methylprednisolone treatment for 15 days at a lower dose, none of those effects were observed (data not shown). 

The increased antioxidant potential induced by short-term administration of methylprednisolone might represent a mechanism of protection against ROS generation after exposure to corticosteroids. ROS can be generated as a consequence of the intracellular metabolism of foreign compounds, toxins, or drugs by the cytochrome P450 monooxygenase system, as well as because of exposure to environmental factors, such as excessive iron salts or UV irradiation.^(^ ^20^ ^)^ Intracellular antioxidants, cell membranes, and extracellular fluids can be upregulated and mobilized in order to neutralize excessive and inappropriate ROS formation. To provide extracellular antioxidant defense mechanisms, respiratory tract epithelial cells synthesize and secrete various antioxidant enzymes, such as extracellular forms of superoxide dismutase^(^ ^21^ ^)^ and glutathione peroxidase,^(^ ^22^ ^)^ as well as several metal-binding proteins (e.g., transferrin and ceruloplasmin) that minimize the involvement of transition metal ions (e.g., iron and copper) in oxidative reactions.^(^ ^21^ ^)^ In addition, the extracellular epithelial lining fluid also contains various non-enzymatic antioxidant systems, including vitamin C (ascorbate) and vitamin E (alpha-tocopherol).^(^ ^23^ ^)^ The TRAP assay employed in the current study is widely used,^(^ ^10^ ^,^ ^14^ ^,^ ^24^ ^)^ and it mostly measures non-enzymatic water-soluble antioxidants, such as glutathione, ascorbic acid, and uric acid. The measurement of all of these antioxidants is essential for assessing antioxidant status. However, the number of different antioxidants in biological samples makes it difficult to measure each separately. In addition, the possible interaction among different antioxidants can make measurements of individual antioxidants less representative than is the overall antioxidant status.^(^ ^24^ ^)^

Our results corroborate those of previous studies, suggesting that short-term administration of corticosteroids is protective against oxidative injury in different tissues in experimental models.^(^ ^24^ ^)^ The short-term administration of prednisolone and dexamethasone has been shown to inhibit ROS generation in platelets, and there is evidence that corticosteroids also inhibit oxidative phosphorylation.^(^ ^4^ ^)^ In contrast, we found that 30 days of methylprednisolone treatment increased LPO levels. The chemiluminescence assay is the easiest method and can be applied to crude biological extracts. Although its specificity has been questioned,^(^ ^25^ ^)^ this particular assay is widely used for ex vivo and in vitro measurements,^(^ ^10^ ^)^ and it is accepted as an empirical window for the examination of the complex process of LPO.^(^ ^25^ ^)^ However, the imbalance between production of ROS and antioxidant defenses in the body is called oxidative stress, which has major health implications.^(^ ^19^ ^)^ If there are too many ROS or too few antioxidants for protection, oxidative stress develops, which can cause permanent damage.^(^ ^26^ ^)^ Although the differences were less than significant, we found that long-term administration of a corticosteroid induced a decrease in TRAP levels and an increase in LPO levels, suggesting that oxidative stress occurred.

One of the earliest and most important components of tissue injury after reperfusion of ischemic organs is ROS production. The major ROS include the superoxide radical, the hydroxyl radical, and hydrogen peroxide. ROS-induced injury targets proteins, enzymes, nucleic acids, cytoskeleton, cell membranes, and lipid peroxides, resulting in decreased mitochondrial function and LPO.^(^ ^27^ ^)^ The damage caused by ROS leads to the loss of microvascular integrity and decreased blood flow. The pathogenesis of the various forms of lung injury has been shown to involve peroxidative breakdown of polyunsaturated fatty acids (due to the effects on membrane function); inactivation of membrane-bound receptors and enzymes; and increased tissue permeability.^(^ ^28^ ^)^ There is increasing evidence that aldehydes, which are generated endogenously during the LPO process, are involved in many of the pathophysiological events associated with oxidative stress in cells and tissues.^(^ ^29^ ^)^ In addition to their cytotoxic properties, lipid peroxides have been increasingly recognized as being important in signal transduction for a number of important events in the lung inflammatory response.^(^ ^30^ ^)^ The oxidative pathway was reported to play a significant role in the etiology of remote lung injury in a rabbit model of hepatoenteric ischemia-reperfusion, as well as in other animal models.^(^ ^31^ ^)^

It is important to emphasize that, by choosing two different administration regimens of methylprednisolone (acute and chronic), we sought to simulate the parenteral administration of high doses, which might be warranted in emergencies, such as in severe acute asthma, and a moderate oral dose, which is used under less urgent circumstances in humans. It should be borne in mind that drug metabolism is more rapid in small animals than in humans, and larger doses are therefore necessary.^(^ ^32^ ^)^ Nevertheless, the fact that we used different drug dose regimens in the two treatments represents a limitation of the present study, because it constitutes a confounding variable.

In conclusion, our results suggest that the acute use of corticosteroids is beneficial to lung tissue, whereas their chronic use is not. In addition, we found that acute administration of methylprednisolone increased antioxidant levels in the lung tissue in rats, which is an important finding, considering the use of this medication in acute events and in lung transplantation. Conversely, the negative effect that chronic treatment with methylprednisolone has on LPO might play a role in the mechanisms of the adverse effects involved in pathological conditions associated with the chronic use of glucocorticoids. Future studies using rat models of ischemia/reperfusion injury in lungs might elucidate the differences between acute and chronic use of corticosteroids, in terms of the mechanisms by which they act on a pathological condition.
