# Supplementary material for: Effects of acute and chronic administration of methylprednisolone on oxidative stress in rat lungs
Source: J Bras Pneumol. 2014 May-Jun;40(3):238–43. doi: 10.1590/S1806-37132014000300006 (PMC4109195; doi:10.1590/S1806-37132014000300006)
Supplement: Translation

# Translation: Efeitos da administração aguda e crônica de metilprednisolona no estresse oxidativo em pulmões de ratos*

**Authors:** Ronaldo Lopes Torres, Iraci Lucena da Silva Torres, Gabriela Laste, Maria Beatriz Cardoso Ferreira, Paulo Francisco Guerreiro Cardoso, Adriane Belló-Klein

**Affiliations:** Hospital Divina Providência, Porto Alegre (RS) Brasil; Departamento de Farmacologia, Instituto de Ciências Básicas da Saúde, Universidade Federal do Rio Grande do Sul, Porto Alegre (RS) Brasil; Instituto de Ciências Básicas da Saúde, Universidade Federal do Rio Grande do Sul, Porto Alegre (RS) Brasil; Departamento de Farmacologia, Instituto de Ciências Básicas da Saúde, Universidade Federal do Rio Grande do Sul, Porto Alegre (RS) Brasil; Departamento de Cardiopneumologia, Disciplina de Cirurgia Torácica, Instituto do Coração, Hospital das Clínicas, Faculdade de Medicina, Universidade de São Paulo, São Paulo (SP) Brasil; Departamento de Fisiologia, Instituto de Ciências Básicas da Saúde, Universidade Federal do Rio Grande do Sul, Porto Alegre (RS) Brasil

**Keywords:** Pulmão, Metilprednisolona, Glucocorticoides, Peroxidação de lipídeos, Elementos de resposta antioxidante

## Abstract

**Objetivo:**

Determinar os efeitos da administração aguda e crônica de metilprednisolona no estresse oxidativo, por meio da quantificação da peroxidação lipídica (POL) e do potencial antioxidante reativo total (PART), em pulmões de ratos.

**Métodos:**

Quarenta ratos Wistar foram divididos em quatro grupos: tratamento agudo, com ratos recebendo uma dose única de metilprednisolona (50 mg/kg i.p.); controle agudo, com ratos recebendo injeção unida de salina; tratamento crônico, com ratos recebendo metilprednisolona v.o. na água do bebedouro (6 mg/kg por dia durante 30 dias; e controle crônico, com ratos recebendo água de bebedouro normal).

**Resultados:**

Os níveis de PART foram significativamente maiores no grupo tratamento agudo que no grupo controle agudo, sugerindo uma melhora do sistema de defesa pulmonar. Os níveis de POL foram significativamente maiores no grupo tratamento crônico que no grupo controle crônico, indicando dano oxidativo no tecido pulmonar.

**Conclusões:**

Nossos resultados sugerem que o uso agudo de corticoides foi benéfico aos tecidos pulmonares, enquanto seu uso crônico não o foi. O uso crônico de metilprednisolona parece aumentar os níveis pulmonares da POL.

## Introdução

Os corticosteroides são amplamente usados em uma vasta gama de doenças respiratórias, como a asma, a rinite alérgica e a DPOC.^(^ ^1^ ^)^ Observou-se que o tratamento agudo com corticosteroides pode suprimir processos inflamatórios e a produção de espécies reativas de oxigênio (ERO).^(^ ^2^ ^)^ Em um estudo recente,^(^ ^3^ ^)^ demonstrou-se que a administração de dexametasona reduz a produção de malondialdeído no tecido pulmonar após a lesão de isquemia/reperfusão e protege os níveis celulares de enzimas antioxidantes. Além disso, demonstrou-se que a administração de prednisolona ou dexametasona em curto prazo inibe a geração de ERO em plaquetas, e há evidências de que os esteroides inibem a fosforilação oxidativa.^(^ ^4^ ^)^ Sugeriu-se que o uso de baixas doses de corticosteroides (1-2 mg/kg por dia) em longo prazo pode beneficiar os pulmões e reduzir o risco de efeitos colaterais sistêmicos em pacientes com síndrome da angústia respiratória aguda,^(^ ^5^ ^)^ ao passo que a administração aguda de altas doses de corticosteroides não traz nenhum benefício a esses pacientes.^(^ ^6^ ^)^

O tratamento crônico com corticosteroides pode induzir vários sintomas e sinais (efeitos colaterais), tais como obesidade de tronco, edema facial ("fácies de lua cheia"), estrias cutâneas, hirsutismo, catarata, osteoporose, miopatia, diabetes mellitus, imunossupressão e doenças cardiovasculares.^(^ ^7^ ^)^ O uso excessivo de corticosteroides pode também induzir a superprodução de ERO pelas células endoteliais.^(^ ^8^ ^)^

Sabe-se bem que os corticosteroides têm efeitos anti-inflamatórios, alguns dos quais podem ser mediados por ERO, que são produtos de processos metabólicos normais nas células. As principais fontes de ERO são vazamentos na cadeia de transporte de elétrons nas mitocôndrias e no retículo endoplasmático. Outra importante fonte de ERO é uma NADH/NADPH oxidase presente na membrana celular. Em baixas concentrações, as ERO atuam como mediadores fisiológicos de respostas celulares e reguladores de expressão gênica.^(^ ^4^ ^)^ O desequilíbrio entre a produção de ERO e as defesas antioxidantes leva ao estresse oxidativo.^(^ ^9^ ^)^ O estresse oxidativo foi considerado um importante fator patológico em doenças pulmonares, neurodegenerativas e autoimunes, bem como em doenças metabólicas, câncer e envelhecimento.^(^ ^10^ ^-^ ^12^ ^)^ Sabe-se bem que as ERO geram uma cascata bioquímica, produzindo peroxidação lipídica (POL), oxidação de proteínas, danos ao DNA e morte celular, que podem contribuir para a ocorrência de patologias relacionadas com um aumento acentuado de ERO e outros radicais livres,^(^ ^13^ ^)^ como por exemplo a lesão pulmonar induzida por isquemia/reperfusão.^(^ ^14^ ^)^ Portanto, as ERO desempenham um papel crucial na cascata de eventos que levam à insuficiência pulmonar. 

Levando em conta o exposto acima, realizamos o presente estudo com o objetivo de determinar o efeito da administração aguda e crônica de metilprednisolona sobre o estresse oxidativo. Para isso, quantificamos a POL e o potencial antioxidante reativo total (PART) em pulmões de ratos.

## Métodos

Quarenta ratos Wistar machos adultos (idade: 60 dias; peso: 200-250 g) que nunca haviam sido submetidos a nenhum tipo de experimento foram aleatoriamente divididos por peso e alojados em grupos de cinco em gaiolas caseiras de polipropileno (49 × 34 × 16 cm). Todos os animais foram mantidos em ciclo claro-escuro de 12 h (luzes acesas das 7h00 às 19h00) em um ambiente com temperatura controlada (22 ± 2°C) e água e ração à vontade. Todos os experimentos e procedimentos foram aprovados pelo comitê institucional de tratamento e uso de animais e foram realizados em conformidade com as diretrizes brasileiras para o uso de animais em pesquisa (Lei nº 11.794) e com diretrizes internacionais. Fizemos grande esforço para minimizar o sofrimento dos animais e diminuir fontes externas de dor e desconforto, bem como para usar apenas o número de animais necessário para produzir dados científicos confiáveis. 

Usamos succinato sódico de metilprednisolona (Solu-Medrol^(r)^; Pharmacia, Nova Iorque, NY, EUA). O pó liofilizado (500 mg) foi dissolvido em 8 mL de solução salina a 0,9%. A solução da droga foi preparada imediatamente antes de sua administração. 

No experimento com tratamento agudo, os animais foram divididos em dois grupos de 10 animais cada. Os ratos em um dos grupos (o grupo de tratamento agudo) receberam uma única injeção (i.p.) de metilprednisolona (50 mg/kg) em um volume de 1 mL/kg da solução, ao passo que os do outro grupo (o grupo de controle agudo) receberam injeção i.p. do mesmo volume de solução salina. 

No experimento com tratamento crônico, os animais foram divididos em dois grupos de 10 animais cada. Os ratos em um dos grupos (o grupo de tratamento crônico) receberam metilprednisolona (6 mg/kg por dia v.o.) na água do bebedouro durante 30 dias, ao passo que os do outro grupo (o grupo de controle crônico) receberam apenas água do bebedouro. Cada 500 mL da água do bebedouro continha 31 mg de succinato sódico de metilprednisolona (0,0625 mg/mL). Considerando-se um consumo médio de 25 mL/dia por rato, cada rato do grupo de tratamento crônico consumiu 1,56 mg de metilprednisolona por dia. 

Vinte e quatro horas após a administração aguda ou no fim do período de tratamento crônico, os animais foram mortos por decapitação. Os pulmões foram extraídos e congelados por meio de imersão em nitrogênio líquido. As amostras foram armazenadas a −80°C até a análise. Os pulmões foram pesados e homogeneizados a 1:5 p/v em líquido gelado (1,15% de KCl e 20 mmol/L de fluoreto de fenilmetilsulfonila), por meio de um homogeneizador Ultra-Turrax (IKA, Toronto, ON, Canadá). Para remover a fração particulada, os homogeneizados foram centrifugados a 1.000 g durante 20 min a 0-4°C, e o sobrenadante foi usado para os ensaios de POL, PART e conteúdo proteico.^(^ ^15^ ^)^

O nível de PART foi determinado por meio da medição da intensidade de quimioluminescência do luminol induzida pela termólise de dicloridrato de 2,2'-azobis(2-amidinopropano).^(^ ^16^ ^)^ Os resultados foram expressos em µM de ácido 2-carboxílico-6-hidroxi-2,5,7,8-tetrametilcromano por mg de proteína. A POL foi quantificada por meio de quimioluminescência. O método é altamente sensível e capaz de detectar pequenas quantidades de produtos de peroxidação. A quimioluminescência foi medida em um contador de cintilação líquida com o circuito de coincidência desconectado (LKB Rack Beta Liquid Scintillation Spectrometer 1215; LKB Produkter AB, Bromma, Suécia). As reações foram iniciadas por meio da adição de 3 mmol/L de hidroperóxido de terc-butilo, e os dados foram expressos em contagens por segundo por mg de proteína no homogeneizado.^(^ ^17^ ^)^ Os níveis de proteína foram medidos pelo método de Lowry et al.,^(^ ^18^ ^)^ e a albumina sérica bovina foi usada como padrão. 

Os dados foram estatisticamente avaliados por meio do teste t de Student e estão expressos na forma de média ± ep. Valores de p < 0,05 foram considerados significativos.

## Resultados

Primeiramente avaliamos o efeito do tratamento agudo com metilprednisolona sobre os níveis de PART e POL em pulmões de ratos. Foi observado um aumento significativo (de 20%) dos níveis totais de PART no grupo tratado (p < 0,05; Figura 1). Não houve diferença significativa entre os grupos quanto aos níveis de POL (p > 0,05; Figura 2).

**Figura 1:** Média dos níveis de potencial antioxidante reativo total (PART) nos pulmões de ratos submetidos a administração aguda de metilprednisolona (grupo de tratamento agudo) ou injeção de um volume igual de solução salina (grupo de controle agudo). Trolox: ácido 2-carboxílico-6-hidroxi-2,5,7,8-tetrametilcromano. *p < 0,05, teste t de Student.

**Figura 2:** Média dos níveis de peroxidação lipídica nos pulmões de ratos submetidos a administração aguda de metilprednisolona (grupo de tratamento agudo) ou injeção de um volume igual de solução salina (grupo de controle agudo). QL: quimioluminescência; e cps: contagens por segundo.

Não encontramos nenhuma diferença entre o grupo de tratamento crônico e o grupo de controle crônico no tocante aos níveis totais de PART (p > 0,05; Figura 3). O grau de dano oxidativo pulmonar, medido por meio de quimioluminescência, foi significativamente (38%) maior no grupo de tratamento crônico do que no grupo de controle crônico (p < 0,05; Figura 4). 

**Figura 3:** Média dos níveis de potencial antioxidante reativo total (PART) nos pulmões de ratos submetidos a administração crônica de metilprednisolona oral (grupo de tratamento crônico) ou não (grupo de controle crônico). Trolox: ácido 2-carboxílico-6-hidroxi-2,5,7,8-tetrametilcromano.

**Figura 4:** Média dos níveis de peroxidação lipídica nos pulmões de ratos submetidos a administração crônica de metilprednisolona oral (grupo de tratamento crônico) ou não (grupo de controle crônico). QL: quimioluminescência; e cps: contagens por segundo. *p < 0,05, teste t de Student

## Discussão

As concentrações de antioxidantes nos pulmões podem ser quantificadas por meio da medição do nível de PART.^(^ ^10^ ^,^ ^14^ ^)^ A concentração relativa de antioxidantes determina a capacidade antioxidante total do tecido. O nível de PART representa principalmente os antioxidantes não enzimáticos solúveis em água no tecido. Além disso, o nível de POL, que desempenha um papel importante na indução de apoptose e formação de radicais livres,^(^ ^19^ ^)^ é amplamente usado como marcador de estresse oxidativo. 

Os resultados do presente estudo mostram que a duração da corticoterapia altera as respostas do sistema oxidativo nos pulmões de ratos. O tratamento agudo com metilprednisolona induziu um aumento significativo dos níveis de PART nos pulmões de ratos sem quaisquer alterações dos níveis de POL. No entanto, quando o tratamento foi mantido por 30 dias, observou-se um aumento dos níveis de POL sem quaisquer alterações dos níveis de PART, o que aumenta o risco de lesão pulmonar oxidativa. Não obstante, quando os animais foram submetidos ao tratamento com uma dose mais baixa de metilprednisolona durante 15 dias, nenhum desses efeitos foi observado (dados não apresentados). 

O aumento do potencial antioxidante induzido pela administração de metilprednisolona em curto prazo pode representar um mecanismo de proteção contra a geração de ERO após a exposição a corticosteroides. As ERO podem ser geradas em consequência do metabolismo intracelular de compostos estranhos, toxinas ou drogas pelo sistema enzimático do citocromo P450, bem como em virtude da exposição a fatores ambientais, tais como excesso de sais de ferro ou irradiação UV.^(^ ^20^ ^)^ Antioxidantes intracelulares, membranas celulares e fluidos extracelulares podem ser suprarregulados e mobilizados a fim de neutralizar a formação excessiva e inadequada de ERO. Para fornecer mecanismos extracelulares de defesa antioxidante, as células epiteliais do trato respiratório sintetizam e secretam várias enzimas antioxidantes, tais como formas extracelulares de superóxido dismutase^(^ ^21^ ^)^ e glutationa peroxidase,^(^ ^22^ ^)^ bem como várias proteínas de ligação a metais (como por exemplo a transferrina e a ceruloplasmina) que minimizam o envolvimento de íons de metais de transição (como por exemplo o ferro e o cobre) em reações de oxidação.^(^ ^21^ ^)^ Além disso, o fluido de revestimento epitelial extracelular também contém vários sistemas antioxidantes não enzimáticos, incluindo a vitamina C (ascorbato) e a vitamina E (alfa-tocoferol).^(^ ^23^ ^)^ O ensaio de PART empregado no presente estudo é amplamente usado^(^ ^10^ ^,^ ^14^ ^,^ ^24^ ^)^ e mede principalmente antioxidantes não enzimáticos solúveis em água, como a glutationa, o ácido ascórbico e o ácido úrico. A medição de todos esses antioxidantes é essencial à avaliação do estado antioxidante. No entanto, o número de antioxidantes diferentes em amostras biológicas torna difícil medir cada um separadamente. Além disso, a possível interação entre os diferentes antioxidantes pode fazer com que medidas de antioxidantes individuais sejam menos representativas do que o estado antioxidante geral.^(^ ^24^ ^)^

Nossos resultados corroboraram os de estudos anteriores, sugerindo que a administração de corticosteroides em curto prazo protege contra a lesão oxidativa em diferentes tecidos em modelos experimentais.^(^ ^24^ ^)^ Demonstrou-se que a administração de prednisolona e dexametasona em curto prazo inibe a geração de ERO em plaquetas, e há evidências de que os corticosteroides inibem também a fosforilação oxidativa.^(^ ^4^ ^)^ Por outro lado, constatamos que 30 dias de tratamento com metilprednisolona aumentaram os níveis de POL. O ensaio de quimioluminescência é o método mais fácil e pode ser aplicado a extratos biológicos brutos. Embora sua especificidade tenha sido questionada,^(^ ^25^ ^)^ esse ensaio em particular é amplamente usado para medições *ex vivo* e *in vitro*,^(^ ^10^ ^)^ e é aceito como uma janela empírica para o exame do complexo processo de POL.^(^ ^25^ ^)^ O desequilíbrio entre a produção de ERO e as defesas antioxidantes do organismo é chamado estresse oxidativo, que tem grandes implicações para a saúde.^(^ ^19^ ^)^ Se há ERO demais ou muito poucos antioxidantes para a proteção, ocorre estresse oxidativo, que pode causar danos permanentes.^(^ ^26^ ^)^ Embora as diferenças não tenham sido significativas, constatamos que a administração de um corticosteroide em longo prazo induziu uma diminuição dos níveis de PART e um aumento dos níveis de POL, o que sugere a ocorrência de estresse oxidativo. 

Um dos primeiros e mais importantes componentes da lesão tecidual após a reperfusão de órgãos isquêmicos é a produção de ERO. As principais ERO incluem o radical superóxido, o radical hidroxila e o peróxido de hidrogênio. A lesão induzida por ERO atinge proteínas, enzimas, ácidos nucleicos, citoesqueleto, membranas celulares e peróxidos lipídicos, resultando em diminuição da função mitocondrial e POL.^(^ ^27^ ^)^ O dano causado por ERO leva à perda de integridade microvascular e à diminuição do fluxo sanguíneo. Demonstrou-se que a patogênese das várias formas de lesão pulmonar envolve quebra peroxidativa de ácidos graxos poli-insaturados (em virtude dos efeitos sobre a função da membrana); inativação de receptores e enzimas ligados à membrana e aumento da permeabilidade dos tecidos.^(^ ^28^ ^)^ Há cada vez mais evidências de que os aldeídos, que são gerados endogenamente durante o processo de POL, estejam envolvidos em muitos dos eventos fisiopatológicos relacionados com o estresse oxidativo em células e tecidos.^(^ ^29^ ^)^ Além de suas propriedades citotóxicas, os peróxidos lipídicos têm sido cada vez mais reconhecidos como sendo importantes na transdução de sinais em diversos eventos importantes na resposta inflamatória pulmonar.^(^ ^30^ ^)^ ão pulmonar remota em um modelo de isquemia-reperfusão hepatoentérica em coelhos, bem como em outros modelos animais.^(^ ^31^ ^)^

É importante ressaltar que, ao escolher dois regimes diferentes de administração de metilprednisolona (agudo e crônico), buscamos simular a administração parenteral de doses elevadas, que se pode justificar em casos de emergência, como na asma aguda grave, e uma dose oral moderada, que é usada em circunstâncias menos urgentes em seres humanos. Deve-se ter em mente que o metabolismo da droga é mais rápido em pequenos animais do que em seres humanos, sendo necessárias, portanto, doses maiores.(32) No entanto, o fato de termos usado diferentes doses de medicamentos nos dois tratamentos representa uma limitação do presente estudo, pois constitui uma variável de confusão. 

Em suma, nossos resultados sugerem que o uso agudo de corticosteroides é benéfico para o tecido pulmonar, ao passo que o uso crônico não o é. Além disso, constatamos que a administração aguda de metilprednisolona aumentou os níveis de antioxidantes no tecido pulmonar de ratos. Trata-se de um achado importante em virtude do uso desse medicamento em eventos agudos e no transplante pulmonar. Por outro lado, o efeito negativo que o tratamento crônico com metilprednisolona tem sobre a POL pode desempenhar um papel nos mecanismos dos efeitos adversos envolvidos em patologias relacionadas com o uso crônico de glucocorticoides. Futuros estudos com modelos de lesão de isquemia/reperfusão em pulmões de ratos podem elucidar as diferenças entre o uso agudo e crônico de corticosteroides no que tange aos mecanismos pelos quais atuam em certas patologias.
